# A Cortical-Inspired Contour Completion Model Based on Contour Orientation and Thickness

**DOI:** 10.3390/jimaging10080185

**Published:** 2024-07-31

**Authors:** Ivan Galyaev, Alexey Mashtakov

**Affiliations:** 1 V. A. Trapeznikov Institute of Control Sciences of RAS, Moscow 117997, Russia; 2 Ailamazyan Program Systems Institute of RAS, Pereslavl-Zalessky 152021, Russia

**Keywords:** visual cortex, contours thickness, similarity group, orientation, scale, sub-Riemannian geodesics, optimal control

## Abstract

An extended four-dimensional version of the traditional Petitot–Citti–Sarti model on contour completion in the visual cortex is examined. The neural configuration space is considered as the group of similarity transformations, denoted as M=SIM(2). The left-invariant subbundle of the tangent bundle models possible directions for establishing neural communication. The sub-Riemannian distance is proportional to the energy expended in interneuron activation between two excited border neurons. According to the model, the damaged image contours are restored via sub-Riemannian geodesics in the space *M* of positions, orientations and thicknesses (scales). We study the geodesic problem in *M* using geometric control theory techniques. We prove the existence of a minimal geodesic between arbitrary specified boundary conditions. We apply the Pontryagin maximum principle and derive the geodesic equations. In the special cases, we find explicit solutions. In the general case, we provide a qualitative analysis. Finally, we support our model with a simulation of the association field.

## 1. Introduction

A mathematical description of the functioning of the human body is a pressing problem in the modern world. Particularly, the specification of cerebration and neuron operation is of particular interest. In this paper, we model the visual information processing by the visual cortex. The complete mechanism of the visual signal processing is not fully studied; however, there is a profound understanding [1] of how image processing is carried out via the information accumulated in light-sensitive receptors, bipolar and ganglion cells of the eye retina. Such information includes the spatial coordinates of the image.

After the retina, the visual signal passes through LGN cells of the thalamus and arrives in the visual cortex. The visual cortex has a multi-layered organization and consists of billions of neural cells. Neurons are connected in a complex network, which is extremely difficult to analyze due to the huge number of elements and even more connections between them. The direct simulation approach to modeling such systems faces inevitable obstacles. However, there are some fundamental principles that are used in network configuration, e.g., the principle of minimum energy spent on establishing communication between two excited neurons of the network. A promising direction for studying such complex systems is to understand such principles and propose simple mathematical models based on these principles. Further mathematical analysis of such models can deepen the understanding of the original systems.

In [2], a mathematical model based on scale space theory was proposed to describe the primary processing mechanism. The model is based on the properties of Gaussian kernels and their derivatives as regularized differential operators, as well as solutions to the linear diffusion equation. The model is supported by experimental study [3], where the shape of the receptive fields (RFs) of bipolar and ganglion cells was established. The authors concluded that RFs are mathematically well approximated by filter profiles based on the Gaussian kernel and Gaussian derivatives. Then, their functioning can be represented as the action of a filter on the input signal (convolution of two functions).

In [4], Marr put forward the idea that retinal ganglion cells perform a convolution capable of extracting qualitative breaks encoded in the signal. Marr also posed that higher levels of visual processing are rooted in this first level of morphological organization of the retinal image, which he called the 2D-primal sketch. He discovered that the convolution of a signal with a receptive profile is a wavelet analysis of the signal, i.e., a spatially localized multiscale Fourier analysis capable of detecting discontinuities. An alternative transformation tool can be an internal compression of the image in accordance with its geometric structure. There is Marr’s hypothesis that the image can be reconstructed from its different-scale edges. This contour reconstruction is extremely accurate as only finer details such as textures are smoothed out. In this case, a problem arises with the origin of the breaks. Discontinuities that are robust to large-scale changes can be mistaken for the edges of external objects. Therefore, compression of visual information, i.e., information limitation, is identified with morphological analysis, i.e., geometric constraint.

Further research required a more comprehensive approach. Hubel and Wiesel [5] understood the principles of the primary visual cortex V1 processing. They showed that some parts of the brain react not only to the spatial position of the visible image, but also to its orientation in space. They found that the receptive fields of V1 neurons were elongated rather than rounded. This indicated the ability of V1 cells to detect contour segments with different orientations throughout the image. Mathematically, the operation of V1 cells can be modeled as lifting a two-dimensional input image into an expanded space of positions and orientations.

Even though V1 is physically a two-dimensional neural layer, it implements more than two degrees of freedom. Hubel [1] called the difference between physical and abstract dimensions the grafting of variables. In his pioneering work [6], Hoffmann introduced differential fiber bundles to describe the visual cortex. Here, the base of the fiber bundle represents the retinal plane and the fibers represent the engrafted variables. Further development of this model was performed by Petitot and Tondut [7] (see also the honorable work [8] by Petitot). They described V1 cells as a fiber bundle equipped with a contact structure, and the neural long-range connections were identified as integral curves in the Heisenberg group. In their model, contour completion is carried out along integral curves by minimizing a suitable length functional.

Petitot [8] described the biological functioning of the visual cortex V1 as a sub-Riemannian (SR) structure on the Heisenberg group. Citti and Sarti [9] took into account the nature of the orientation angle and proposed the SR structure on the Lie group $SE(2)=R^{2}\times SO(2)$. The roto-translation group SE(2) models the configuration space of V1 neurons as the space of $R^{2}$ positions and SO(2) orientations. In this model, the reconstruction of a hidden contour occurs by minimizing the excitation energy of neurons that perceive visual information. Such a process is interpreted as the action of the hypoelliptic diffusion operator studied in [10,11,12]. Then, the reconstructed parts of the contour are the SR length minimizers on SE(2). The exact expression of the minimizers was found in [13]. Such curves are used to render images [14] and to explain some visual illusions [15,16].

Modern computer vision is actively developing based on the principles of biological systems. They are used for image analysis tasks such as enhancement, segmentation, shading, and feature detection. In [17,18], the authors provided a mathematical background for image processing in an extended space of positions and orientations. Salient lines are tracked by SR length minimizers (see [19,20]) or by optimal trajectories in modified models [21,22,23,24], where the spatial propagation along the minimizers is restricted to avoid cusp points and to normalize the curvature of the detected salient lines.

The classic Petitot–Citti–Sarti model has been widely developed. It is the cornerstone of an entire scientific direction—neuromathematics of vision [25,26]. This model was modified in subsequent works by many authors (e.g., [27,28,29,30,31,32,33,34,35]), taking into account some aspects of the physiology of vision and based on the needs in the field of image processing. A recent work [36] showed an overview of different models.

The work [27] bridged the classic Petitot–Citti–Sarti model with the edge co-occurrence statistics in natural images. The model’s applicability for the association field construction was studied in [28], where the authors showed that the boundary conditions connected by the SR geodesic with cuspless planar projection match the criteria of good continuation. In [29], the authors modified the model to account for the spherical nature of the retina. Later, in [30], this spherical model was extended by considering the non-uniform distribution of photoreceptors on the retina. A link to the well-known Bresloff–Cowan spherical model [31] of V1 hypercolumns was studied in [32]. A link to the widely used Wilson–Cowan equations of neural dynamics was studied in [37]. In [33], a four-dimensional model accounting for the contour curvature was studied. In [38], the authors proposed a five-dimensional model considering the duration and velocity of visual stimuli. Another five-dimensional extension was proposed in [39], where orientation, frequency, and phase selective behavior of the V1 simple cells are analyzed. Based on this model, a contour completion method was developed in [40]. A semidiscrete modification and its application to image processing was studied in [34].

In [35], the authors expanded their classic model by adding a scale parameter and introducing a symplectic structure to describe the structure of neural connectivity. In the current work, we take this model as a basis and develop it by explicitly introducing the length functional and considering the problem of finding length minimizers between arbitrarily specified boundary conditions.

**Figure 1:**
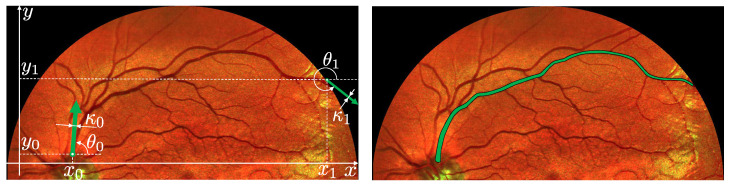
Finding blood vessels (salient lines) in the fundus photography of the human retina. Specifications: *x*, *y* are spatial coordinates, θ is the orientation, and $\kappa =e^{\sigma }$ is the thickness of lines.

**Figure 2:**
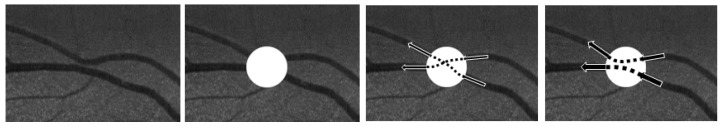
Restoration of image contours. From left to right: original image; corrupted image (the damaged area is a white disc); recovering contours via the classical model (sub-Riemannian geodesics in SE(2)); restoration via geodesics in SIM(2), taking into account the thickness of contours.

Neurophysiological studies show that spatial hypercolumns also accumulate secondary information about the visible image, such as ocular dominance [41], contour curvature [42,43], contour thickness (scale) [44], and other features. It is known that neurons (simple cells) have different sizes in different areas of the visual cortex. Hence, from V1 to V2, we find simple cells sensitive to objects of different scales (see, e.g., Figure 37 in [25]). In this paper, inspired by [35], we consider a four-dimensional model, which is an extension of the classic Petitot–Citti–Sarti model by adding the contour thickness parameter. The configuration space of neurons is interpreted as a group of similarity transformations M=SIM(2). The left-invariant distribution [45] of the tangent subspaces models the possible directions of establishing a neural connection. The sub-Riemannian distance is proportional to the energy expended in interneuron activation between two excited border neurons. According to the model, the contours of the damaged image are restored using sub-Riemannian geodesics in the space *M* of positions, orientations and thicknesses (scales). This extension is also intended for image processing tasks to find salient lines (see Figure 1), and to restore damaged image contours (see Figure 2). Image enhancement via left-invariant evolution in the SIM(2) group is studied in [46].

In this paper, we consider the problem of SR geodesics in SIM(2). In Section 2, we formulate the model and state the sub-Riemannian problem. In Section 3, we prove the complete controllability of the system and the existence of optimal controls. Then, in Section 4, we apply a necessary optimality condition, the Pontryagin maximum principle (PMP), and study the Hamiltonian PMP system. We obtain an explicit expression for the abnormal geodesics and provide a qualitative analysis of the Hamiltonian system for normal geodesics. In Section 5, we discuss the boundary value problem. In Section 6, we provide a simulation to construct the association field using the sub-Riemannian geodesics in our problem.

The main contributions of our research are the following:

- An optimal control formulation of the contour completion problem; see (15) and (16).
- A proof of well-posedness of the geodesic problem in *M*; see Theorem 1.
- An explicit expression of minimal abnormal geodesics; see Theorem 2.
- An explicit expression of a special case of normal geodesics; see Theorem 3.
- Asymptotic behavior of normal geodesics in the general case; see Theorem 4.

## 2. Problem Formulation

The classic works of Petitot, Citti, and Sarti [8,9] present a model of V1 as a three-dimensional Lie group SE(2) of positions and orientations. In their model, horizontal long-range connections between cells of V1 are represented by smooth curves adhering to a nonholonomic constraint: the curves must be tangent to the distribution $\tilde{\Delta }=\mathrm{Ker}\tilde{\omega }$, where $\tilde{\omega }\in \Lambda^{1}SE(2)$ is a given left-invariant differential one-form. Through this approach, a horizontal connection between two neurons is established based on the principle of minimum energy spent on its creation. This leads to the natural modeling of the space V1 by the sub-Riemannian manifold $\left(SE(2),\tilde{\Delta },\tilde{g}\right)$, where the metric $\tilde{g}$ specifies the distance encoding the expended energy. According to the model, the visual system performs contour completion (restoration of a corrupted or partially hidden from observation contour) by finding a sub-Riemannian length minimizer between two configurations on the boundary of the damaged area (see Figure 2).

In the consequent work [44], the same authors introduced a new variable σ and defined a symplectic structure in the extended space SIM(2) of positions, orientations and scales. The symplectic structure generates nonholonomic constraints for establishing a long-range neural connection. Note that the explicit form of a metric encoding the energy spent to create the connection was not considered in [44]. In our work, we explicitly present this sub-Riemannian metric and formulate an optimal control problem for finding length minimizers. In [44], the authors presented special types of integral curves with a fixed parameter of scale or orientation, which correspond to constant controls in our model. We are also motivated by image analysis applications, where the thickness of contours varies during tracking (see Figure 1).

According to the model, the contour completion mechanism by V1 is invariant under parallel translations, rotations, and scaling of the image on the retina. Such transformations constitute the group of orientation-preserving similarity transformations on the plane

(1) $$SIM(2)=\left\{q=\left.\left(\begin{array}{ccc} e^{\sigma }\cos\theta & -e^{\sigma }\sin\theta & x\\ e^{\sigma }\sin\theta & e^{\sigma }\cos\theta & y\\ 0 & 0 & 1 \end{array}\right)\;\right|\;\left(x,y\right)\in R^{2},\theta \in S^{1},\sigma \in R\right\}.$$

The retinal plane is a homogeneous space of the Lie group SIM(2), which acts transitively on it. Thus, SIM(2) models the configuration space of simple cells V1.

Now, we explain the lifting of an observable image from the retinal plane to the group SIM(2), which represents V1. The set of receptive profiles of V1 simple cells over a retinal point is formed from the “mother” Gabor function (see [44]),

(2) $$G_{\left(0,0\right)}\left(x,y\right)=e^{-(x^{2}+y^{2})}\cos2y,$$

by rotations on an angle θ, and dilations on $e^{\sigma }$:

(3) $$G_{\left(\theta ,\sigma \right)}\left(x,y\right)=e^{-2\sigma }e^{-\left(x_{\theta }^{2}+y_{\theta }^{2}\right)}\cos2y_{\theta },$$

where

(4) $$x_{\theta }=e^{-\sigma }\left(x\cos\theta +y\sin\theta \right),\;y_{\theta }=e^{-\sigma }\left(-x\sin\theta +y\cos\theta \right).$$

The lifted image $O\left(x,y,\theta ,\sigma \right):SIM(2)\to R^{+}$ is obtained by probing the observable image $I\left(x,y\right):R^{2}\to R^{+}$ on the retinal plane with a family of two parametric Gabor filters,

(5) $$O\left(x,y,\theta ,\sigma \right)=\left(I*G_{\left(\theta ,\sigma \right)}\right)\left(x,y\right)=\int_{R^{2}}I\left(\xi ,\eta \right)G_{\left(\theta ,\sigma \right)}\left(x-\xi ,y-\eta \right)d\;\xi d\;\eta .$$

A selection from all different cells in a fiber is performed by maximum response selection

(6) $$\left(\bar{\theta },\bar{\sigma }\right)={\mathrm{argmax}}_{\theta \in S^{1},\sigma \in R}O\left(x,y,\theta ,\sigma \right).$$

The values $\left(\bar{\theta },\bar{\sigma }\right)$ are actual values of engrafted variables θ and σ associated with a retinal point (x,y). Such values are used as boundary conditions for contour completion problem that we formulate at the end of this paragraph as an optimal control problem.

Beforehand, we explain a nonholonomic constraint on a long-range (horizontal) neural connection. A horizontal connection is modeled by a smooth curve tangent to the distribution Δ=Kerω, where the one-form $\omega \in \Lambda^{1}SIM(2)$ is given by (see [44])

(7) $$\omega =e^{-\sigma }\left(-\sin\theta \;d\;x+\cos\theta \;d\;y\right).$$

Notice that the distribution Δ is given by

(8) $$\Delta =\mathrm{span}\left(X_{1},X_{3},X_{4}\right)=u_{1}X_{1}+u_{3}X_{3}+u_{4}X_{4},\;u_{i}\in R,$$

where $X_{i}$ are basis left-invariant vector fields on SIM(2)

(9) $$\begin{array}{ccc} & & X_{1}\left(q\right)=L_{q*}{\left.\frac{\partial }{\partial x}\right|}_{\mathrm{Id}}=e^{\sigma }\left(\cos\theta \frac{\partial }{\partial x}+\sin\theta \frac{\partial }{\partial y}\right), \end{array}$$

(10) $$\begin{array}{ccc} & & X_{2}\left(q\right)=L_{q*}{\left.\frac{\partial }{\partial y}\right|}_{\mathrm{Id}}=e^{\sigma }\left(-\sin\theta \frac{\partial }{\partial x}+\cos\theta \frac{\partial }{\partial y}\right), \end{array}$$

(11) $$\begin{array}{ccc} & & X_{3}\left(q\right)=L_{q*}{\left.\frac{\partial }{\partial \theta }\right|}_{\mathrm{Id}}=\frac{\partial }{\partial \theta }, \end{array}$$

(12) $$\begin{array}{ccc} & & X_{4}\left(q\right)=L_{q*}{\left.\frac{\partial }{\partial \sigma }\right|}_{\mathrm{Id}}=\frac{\partial }{\partial \sigma }, \end{array}$$

where Id is a unit element, and $L_{q}h=qh$ is the left translation on SIM(2) (see Appendix A).

By a horizontal curve, we call a Lipschitz curve tangent to Δ at almost every point

(13) $$\gamma \left(t\right):\left[0,T\right]\to SIM(2),\;\dot{\gamma }\left(t\right)=u_{1}\left(t\right)X_{1}\left(\gamma \left(t\right)\right)+u_{3}\left(t\right)X_{3}\left(\gamma \left(t\right)\right)+u_{4}\left(t\right)X_{4}\left(\gamma \left(t\right)\right),$$

where $u_{i}\left(t\right)\in L^{\infty }\left(\left[0,T\right],R\right)$.

We construct the sub-Riemannian metric by requiring that $X_{1}$, $X_{3}$, and $X_{4}$ be orthogonal. Thus, the length of a horizontal curve is given by

(14) $$l\left(\gamma \right)=\int_{0}^{T}∥\dot{\gamma }\left(t\right)∥d\;t=\int_{0}^{T}\sqrt{u_{1}^{2}\left(t\right)+\alpha^{2}u_{3}^{2}\left(t\right)+\beta^{2}u_{4}^{2}\left(t\right)}d\;t,$$

where the parameters α>0, β>0 are coefficients of the sub-Riemannian metric that encode the balance between penalties for motion in a plane along the contour and changing its orientation and thickness. Further, for simplicity, we consider the model case α=β=1.

Any horizontal curve γ(t) of positive length can be reparameterized by arc length $∥\dot{\gamma }\left(t\right)∥=1$ (see Lemma 3.15 in [47]). Thus, the problem of length minimization l(γ)→min is equivalent to time-optimal problem T→min.

Finally, we formulate a contour completion problem as the optimal control problem. Consider the following control system:

(15) $$\left\{\begin{array}{c} \dot{x}=u_{1}e^{\sigma }\cos\theta ,\\ \dot{y}=u_{1}e^{\sigma }\sin\theta ,\\ \dot{\theta }=u_{3},\\ \dot{\sigma }=u_{4}, \end{array}\right.\;\begin{array}{c} (x,y,\theta ,\sigma )=q\in SIM(2),\\ (u_{1},u_{3},u_{4})\in U,\\ U=\left\{\left.\left(u_{1},u_{3},u_{4}\right)\in R^{3}\;\right|\;u_{1}^{2}+u_{3}^{2}+u_{4}^{2}\leq 1\right\}. \end{array}$$

For given boundary conditions $q_{0}$, $q_{1}\in SIM(2)$, we aim to find the controls $u_{1}\left(t\right)$, $u_{3}\left(t\right)$, $u_{4}\left(t\right)\in L^{\infty }\left(\left[0,T\right],R\right)$, such that the corresponding trajectory q:[0,T]→SIM(2) transfers the system from the initial configuration $q_{0}$ to the final configuration $q_{1}$ by minimum time:

(16) $$q\left(0\right)=q_{0},\;q\left(T\right)=q_{1},\;T=\int_{0}^{T}\;d\;t\to \min.$$

**Remark** **1.** *The problem is invariant under the left action of SIM(2) since the vector fields $X_{1}$, $X_{3}$, and $X_{4}$ are left-invariant. Due to this property, without loss of generality, we set q(0)=Id.*

## 3. Existence of Solutions

When studying Problems (15) and (16), the natural question arises about the existence of an admissible trajectory connecting boundary conditions (16). The control system is called completely controllable if an admissible trajectory exists for any $q_{0}$, $q_{1}\in SIM(2)$. We study the complete controllability of System (15) using the technique of geometric control theory [48].

We have the following nonzero Lie brackets of the controlled vector fields:

(17) $$\left[X_{1},X_{3}\right]=-X_{2},\;\;\left[X_{1},X_{4}\right]=-X_{1},\;\;\left[X_{2},X_{3}\right]=X_{1},\;\;\left[X_{2},X_{4}\right]=-X_{2}.$$

System (15) is symmetric with respect to the controls and it satisfies Hormander condition (i.e., the Lie algebra of the controlled vector fields spans at every point q∈SIM(2) the entire tangent space):

(18) $${\mathrm{Lie}}_{q}\left(X_{1},X_{3},X_{4}\right)=\mathrm{span}\left(X_{1}\left(q\right),\left[X_{3},X_{1}\right]\left(q\right),X_{3}\left(q\right),X_{4}\left(q\right)\right)=T_{q}SIM(2).$$

By Chow–Rashevsky theorem [48], these two conditions plus connectedness of SIM(2) guarantee complete controllability.

Existence of an optimal admissible trajectory that satisfies conditions (16) is ensured by Filippov’s theorem [48,49]. In such a way, we proved the following theorem.

**Theorem** **1.** *Solutions to the optimal control problems (15) and (16) exist for any boundary condition.*

## 4. Pontryagin Maximum Principle

The necessary condition for optimality is given by the Pontryagin maximum principle (PMP). In this section, we apply PMP to our problem (15) and (16).

Let $p\in T_{q}^{*}SIM(2)$. Define the Pontryagin function

(19) $$\begin{array}{ccc} H_{u}\left(p,q\right) & & =\langle p,u_{1}X_{1}\left(q\right)+u_{3}X_{3}\left(q\right)+u_{4}X_{4}\left(q\right)\rangle \\ & & =u_{1}e^{\sigma }\left(p_{1}\cos\theta +p_{2}\sin\theta \right)+u_{3}p_{3}+u_{4}p_{4}, \end{array}$$

where $\left(p_{1},p_{2},p_{3},p_{4}\right)$ are coordinates in $T_{q}^{*}SIM(2)$ corresponding to (x,y,θ,σ) in SIM(2).

PMP states the following. Let u(t), q(t), t∈[0,T] be the optimal control and the corresponding optimal trajectory. Then, there exists a Lipschitz curve p(t) such that $\sum_{i=1}^{4}p_{i}^{2}\left(t\right)\neq 0$ for t∈[0,T] (the non-triviality condition), and the following conditions hold for almost every t∈[0,T]:

1. The Hamiltonian system
  (20) $$\dot{p}\left(t\right)=-\frac{\partial H_{u}}{\partial q}\left(p\left(t\right),q\left(t\right)\right),\;\dot{q}\left(t\right)=\frac{\partial H_{u}}{\partial p}\left(p\left(t\right),q\left(t\right)\right);$$
2. The maximum condition
  (21) $$H_{u(t)}\left(p\left(t\right),q\left(t\right)\right)=\max_{u\in U}\;H_{u}\left(p\left(t\right),q\left(t\right)\right)=H\left(p\left(t\right),q\left(t\right)\right)\geq 0.$$

The function H(p,q) being maximized is called the Hamiltonian. This is the first integral of the Hamiltonian system. The case H=0 is called abnormal, and the case H>0 is called normal. The normal case is reduced to H=1 by time reparameterization.

The Hamiltonian system (20) in our problems (15) and (16) is given by

(22) $$\left\{\begin{array}{c} \dot{x}=u_{1}e^{\sigma }\cos\theta ,\\ \dot{y}=u_{1}e^{\sigma }\sin\theta ,\\ \dot{\theta }=u_{3},\\ \dot{\sigma }=u_{4}, \end{array}\right.\;\left\{\begin{array}{c} {\dot{p}}_{1}=0,\\ {\dot{p}}_{2}=0,\\ {\dot{p}}_{3}=u_{1}e^{\sigma }\left(p_{1}\sin\theta -p_{2}\cos\theta \right),\\ {\dot{p}}_{4}=u_{1}e^{\sigma }\left(-p_{2}\sin\theta -p_{1}\cos\theta \right). \end{array}\right.$$

Natural coordinates for left-invariant systems [45] are given by $h_{i}\left(p,q\right)=\langle p,X_{i}\left(q\right)\rangle$:

(23) $$h_{1}=e^{\sigma }\left(p_{1}\cos\theta +p_{2}\sin\theta \right),\;h_{2}=e^{\sigma }\left(p_{1}\sin\theta -p_{2}\cos\theta \right),\;h_{3}=p_{3},\;h_{4}=p_{4}.$$

The inverse transformation from *h* to *p* is given by

(24) $$p_{1}=e^{-\sigma }\left(h_{1}\cos\theta -h_{2}\sin\theta \right),\;p_{2}=e^{-\sigma }\left(h_{1}\sin\theta +h_{2}\cos\theta \right),\;p_{3}=h_{3},\;p_{4}=h_{4}.$$

The Pontryagin function (19) is expressed in the coordinates (23) as follows:

(25) $$H_{u}=u_{1}h_{1}+u_{3}h_{3}+u_{4}h_{4}.$$

The Hamiltonian system (22) takes the following form in the coordinates (23):

(26) $$\left\{\begin{array}{c} \dot{x}=u_{1}e^{\sigma }\cos\theta ,\\ \dot{y}=u_{1}e^{\sigma }\sin\theta ,\\ \dot{\theta }=u_{3},\\ \dot{\sigma }=u_{4}, \end{array}\right.\;\left\{\begin{array}{c} {\dot{h}}_{1}=u_{3}h_{2}+u_{4}h_{1},\\ {\dot{h}}_{2}=-u_{3}h_{1}+u_{4}h_{2},\\ {\dot{h}}_{3}=-u_{1}h_{2},\\ {\dot{h}}_{4}=-u_{1}h_{1}, \end{array}\right.$$

with the boundary conditions

(27) $$x\left(0\right)=y\left(0\right)=\theta \left(0\right)=\sigma \left(0\right)=0,\;h_{i}\left(0\right)=h_{i0}\in R.$$

The subsystem for the configuration variables *x*, *y*, θ, σ is called the horizontal part, and the subsystem for adjoint variables $h_{i}$ is called the vertical part of the Hamiltonian system.

**Remark** **2.** *The vertical part of the Hamiltonian system (26) can be alternatively derived by computing the Poisson brackets: ${\dot{h}}_{i}=\left\{H_{u},h_{i}\right\}$ (see [47]).*

In PMP formulation for Problems (15) and (16) of searching for a non-trivial (of non-zero length) optimal curve without loss of generality, we choose the arc length parameterization

(28) $$U=\left\{\left.\left(u_{1},u_{3},u_{4}\right)\in R^{3}\;\right|\;u_{1}^{2}+u_{3}^{2}+u_{4}^{2}=1\right\}.$$

Next, we consider two cases: H=0 (the abnormal case), and H=1 (the normal case).

### 4.1. Abnormal Case H=0

The Pontryagin function is given by $H_{u}=u_{1}h_{1}+u_{3}h_{3}+u_{4}h_{4}$. Since the set of admissible controls $U:u_{1}^{2}+u_{3}^{2}+u_{4}^{2}=1$ is symmetric, the maximum condition $H=\max_{u\in U}H_{u}=0$ is satisfied if and only if $h_{1}^{2}+h_{3}^{2}+h_{4}^{2}\equiv 0$. Then, the vertical part of (26) is reduced to

(29) $$\left\{\begin{array}{c} {\dot{h}}_{1}=u_{3}h_{2}=0,\\ {\dot{h}}_{2}=u_{4}h_{2},\\ {\dot{h}}_{3}=-u_{1}h_{2}=0,\\ {\dot{h}}_{4}=0, \end{array}\right.\Rightarrow \left\{\begin{array}{c} u_{1}=u_{3}=0,\\ u_{4}=\pm 1,\\ {\dot{h}}_{2}=u_{4}h_{2}. \end{array}\right.$$

The horizontal part of (26) is reduced to $\dot{x}=\dot{y}=\dot{\theta }=0$, $\dot{\sigma }=u_{4}$. Taking into account the boundary condition x(0)=y(0)=θ(0)=σ(0)=0 leads to the following theorem.

**Theorem** **2.** *Abnormal extremal trajectories have the following form:*

(30) $$x\left(t\right)=y\left(t\right)=\theta \left(t\right)=0,\;\;\;\sigma \left(t\right)=\int_{0}^{t}u_{4}\left(\tau \right)d\;\tau ,$$

*where $u_{4}\left(\cdot \right)$ is an integrable function with values ±1.*

*Abnormal optimal trajectories parameterized by arc length have the following form:*

(31) x(t)=y(t)=θ(t)=0,σ(t)=±t.

The expression (31) of optimal trajectories immediately follows from (30) since, in a time-optimal problem, a motion of a system in opposite directions is not optimal. Therefore, the sign of $u_{4}$ is not changed.

### 4.2. Normal Case H=1

The Pontryagin function $H_{u}=u_{1}h_{1}+u_{3}h_{3}+u_{4}h_{4}$ can be considered as a scalar product $H_{u}=\langle \left(u_{1},u_{3},u_{4}\right),\left(h_{1},h_{3},h_{4}\right)\rangle$. It reaches maximum on the control set $U:u_{1}^{2}+u_{3}^{2}+u_{4}^{2}=1$ when the vector $\left(u_{1},u_{3},u_{4}\right)$ is collinear to $\left(h_{1},h_{3},h_{4}\right)$ and has unit length. Note that, due to the choice of H=1, this implies the following relation for extremal controls:

(32) $$u_{1}=h_{1},\;u_{3}=h_{3},\;u_{4}=h_{4}.$$

The Hamiltonian system takes the form

(33) $$\left\{\begin{array}{c} \dot{x}=h_{1}e^{\sigma }\cos\theta ,\\ \dot{y}=h_{1}e^{\sigma }\sin\theta ,\\ \dot{\theta }=h_{3},\\ \dot{\sigma }=h_{4}, \end{array}\right.\;\left\{\begin{array}{c} {\dot{h}}_{1}=h_{3}h_{2}+h_{4}h_{1},\\ {\dot{h}}_{2}=-h_{3}h_{1}+h_{4}h_{2},\\ {\dot{h}}_{3}=-h_{1}h_{2},\\ {\dot{h}}_{4}=-h_{1}^{2}. \end{array}\right.$$

This system has a first integral: the Hamiltonian *H*. We can find more first integrals by considering right-invariant vector fields $Y_{i}=R_{q*}A_{i}$, $R_{q}h=h\cdot q$, and associated linear on the fibers of the cotangent bundle Hamiltonians $g_{i}=\langle p,Y_{i}\rangle$, $p\in T_{q}^{*}SIM(2)$. Among these four right-invariant Hamiltonians, only two ($g_{1}$, $g_{2}$) are in involution (i.e., the Poisson bracket $\left\{g_{1},H\right\}=\left\{g_{2},H\right\}=\left\{g_{1},g_{2}\right\}=0$) and functionally independent. Thus, we found the following set of first integrals:

(34) $$g_{1}=e^{-\sigma }\left(h_{1}\cos\theta -h_{2}\sin\theta \right),\;g_{2}=e^{-\sigma }\left(h_{2}\cos\theta +h_{1}\sin\theta \right),\;H=h_{1}^{2}+h_{3}^{2}+h_{4}^{2}.$$

To prove Liouville integrability, it is necessary to find four functionally independent first integrals in involution. Three of them we found above. We could not find the remaining first integral. Thus, the question of Liouville integrability remains open.

Now, we focus on the vertical part and describe the coadjoint orbits [50]. Consider the Poisson bivector, which is given by a matrix $P=\left(P_{ij}\right)$ with the components $P_{ij}=\left\{h_{i},h_{j}\right\}$. The structure of Poisson brackets coincides with the structure of Lie brackets (17). Thus, we have only the following nonzero Poisson brackets:

(35) $$\left\{h_{1},h_{3}\right\}=-h_{2},\;\;\left\{h_{1},h_{4}\right\}=-h_{1},\;\;\left\{h_{2},h_{3}\right\}=h_{1},\;\;\left\{h_{2},h_{4}\right\}=-h_{2}.$$

We have $\det P={\left(h_{1}^{2}+h_{2}^{2}\right)}^{2}$; thus rankP=0 if $h_{1}^{2}+h_{2}^{2}=0$, and rankP=4 otherwise.

In the case $h_{1}^{2}+h_{2}^{2}=0$, the coadjoint orbit is zero dimensional. The explicit expression for the extremals are given by the following theorem.

**Theorem** **3.** *For the initial covector values*

(36) $$h_{10}=h_{20}=0,\;h_{30}^{2}+h_{40}^{2}=1,$$

*normal extremal trajectories have the following form:*

(37) $$x\left(t\right)=y\left(t\right)=0,\;\;\theta \left(t\right)=h_{30}\;t,\;\;\sigma \left(t\right)=h_{40}\;t.$$

*They are optimal on a time interval $t\in [0,\frac{\pi }{h_{30}}]$, when $h_{30}\neq 0$; and up to infinity, when $h_{30}=0$.*

**Proof.**  The expression (37) is obtained by integration of the Hamiltonian system (33) with the boundary conditions (27) that, due to the condition (36), is reduced to

(38) $$\dot{x}=\dot{y}=0,\;\dot{\theta }=h_{3},\;\dot{\sigma }=h_{4},\;{\dot{h}}_{1}={\dot{h}}_{2}={\dot{h}}_{3}={\dot{h}}_{4}=0.$$

Optimality for $h_{30}=0$ holds, since the corresponding extremal trajectory is a straight line passing at maximum speed. In the time-optimal problem, this trajectory is optimal, since any other trajectory of the control system requires more time to reach a point on this line.

Optimality for $h_{30}\neq 0$ for $t\in [0,\frac{\pi }{h_{30}}]$ holds for the same reason.

The trajectory for $h_{30}\neq 0$ is not optimal for $t>\frac{\pi }{h_{30}}$, since it has a Maxwell point [45] at $t=\frac{\pi }{h_{30}}$. A Maxwell point is a point where two distinct geodesics meet with the same time. After a Maxwell point, an extremal loses its optimality. A reason for the Maxwell point in our case is periodicity of the angle $\theta \in S^{1}$. Indeed, consider two trajectories with the initial values $h_{1}\left(0\right)=h_{2}\left(0\right)=0$, $h_{3}\left(0\right)=\pm h_{30}$, $h_{4}\left(0\right)=h_{40}$. They reach the same configuration x(t)=y(t)=0, θ(t)=π, $\sigma \left(t\right)=\frac{h_{40}}{h_{30}}\pi$ at $t=\frac{\pi }{h_{30}}$. □

**Remark** **3.** *The abnormal optimal trajectories (31) coincide with normal optimal trajectories (37) when $h_{30}=0$. Thus, they are not strictly abnormal.*

In the general case $h_{1}^{2}+h_{2}^{2}>0$, the coadjoint orbit is four dimensional. We performed a qualitative analysis of the Hamitonian system leading to the following theorem.

**Theorem** **4.** *Any solution to the vertical part corresponding to the initial covector $h_{10}^{2}+h_{20}^{2}>0$, $h_{40}<0$ has the following asymptotic behavior:*

(39) $$\lim_{t\to \infty }h_{1}\left(t\right)=0,\;\;\lim_{t\to \infty }h_{2}\left(t\right)=0,\;\;\lim_{t\to \infty }h_{3}\left(t\right)=h_{31},\;\;\lim_{t\to \infty }h_{4}\left(t\right)=h_{41},\;h_{31}^{2}+h_{41}^{2}=1.$$

**Proof.**  Let $r\left(t\right)=h_{1}^{2}\left(t\right)+h_{2}^{2}\left(t\right)$. Due to system (33), we have

(40) $$\dot{r}\left(t\right)=2h_{4}\left(t\right)r\left(t\right)\;\Rightarrow \;r\left(t\right)=e^{\int_{0}^{t}2h_{4}\left(\tau \right)d\;\tau }r\left(0\right).$$

Now, we estimate the above integral. Due to (33), we have ${\dot{h}}_{4}\left(t\right)=-h_{1}^{2}\left(t\right)\leq 0$. Thus, the function $h_{4}\left(t\right)$ is non-increasing. Together with $h_{4}\left(0\right)=h_{40}<0$, this implies $h_{4}\left(t\right)\leq h_{40}<0$ for all t≥0. Then, the integral of $h_{4}$ decreases indefinitely: $\int_{0}^{t}2h_{4}\left(\tau \right)d\;\tau \leq 2h_{40}t\to -\infty$. Then, the exponential of the integral tends to zero: $e^{\int_{0}^{t}2h_{4}\left(\tau \right)d\;\tau }\to 0$. Thus, we proved $\lim_{t\to \infty }h_{1}\left(t\right)=\lim_{t\to \infty }h_{2}\left(t\right)=0$. It remains to prove the asymptotic behavior of $h_{3}\left(t\right)$ and $h_{4}\left(t\right)$. The Hamiltonian $h_{1}^{2}\left(t\right)+h_{3}^{2}\left(t\right)+h_{4}^{2}\left(t\right)=1$ implies that $h_{4}\left(t\right)$ is bounded from below: $h_{4}\left(t\right)\geq -1$. The boundness and non-increasing of $h_{4}\left(t\right)$ implies $\lim_{t\to \infty }h_{4}\left(t\right)=h_{41}$. The remaining statement $\lim_{t\to \infty }h_{3}\left(t\right)=h_{31}$ follows from the Hamiltonian, since $h_{3}^{2}\left(t\right)\to 1-h_{40}^{2}$. □

Note that the condition $h_{40}<0$ is technical, and we use it in the proof. Based on the numerical experiments, we formulate the following conjecture.

**Hypothesis** **1.** *Any solution to the vertical part corresponding to the initial covector $h_{10}^{2}+h_{20}^{2}>0$ has the following asymptotic behavior:*

(41) $$\lim_{t\to \infty }h_{1}\left(t\right)=0,\;\;\lim_{t\to \infty }h_{2}\left(t\right)=0,\;\;\lim_{t\to \infty }h_{3}\left(t\right)=h_{31},\;\;\lim_{t\to \infty }h_{4}\left(t\right)=h_{41},\;h_{31}^{2}+h_{41}^{2}=1.$$

## 5. Approach to the Boundary Value Problem

A geodesic on a sub-Riemannian manifold is a horizontal curve whose sufficiently short arcs are length minimizers. In the optimal control formulation, SR geodesics are the Pontryagin extremal trajectories. Note that PMP is only a necessary, but not sufficient, condition for optimality. It is an infinite-dimensional analogy of the zero derivative condition when minimizing a smooth function in $R^{n}$. One needs higher-order conditions to find the minimum among all the critical points. The Pontryagin extremals are first-order candidates for being optimal among all admissible trajectories of a control system. Sufficiently short arcs of SR geodesics are optimal, since they satisfy the Legendre condition [48]. An extremal trajectory loses optimality at a so-called cut point [47].

There are two types of Pontryagin extremals, called abnormal and normal. In the previous section, we found an explicit expression for the abnormal extremals and derived the Hamiltonian system (33) for the normal extremals in SIM(2). Note that the set of reachable end conditions for arbitrary time (the attainable set) by the abnormal extremals is a one-dimensional subspace in the four-dimensional space SIM(2). In contrast, the attainable set by the normal extremals is the entire group SIM(2), as we proved in Theorem 1.

By varying the initial value of covector h(0) over the set $C=\{h\in R^{4}\;|\;H=1\}$, we obtain a three-parameter family of the normal extremals. Consider a so-called exponential map Exp(h(0),t):C→SIM(2), which maps the initial covector and the instance of time t>0 to the point γ(t) of the corresponding geodesic. It holds for general sub-Riemannian manifolds that the exponential map is not injective. For example, consider the initial covector h(0)=(0,0,1,0), then the corresponding extremal trajectory is given by θ(t)=Mod(t,2π) (see (33)), which is periodic with a period 2π.

Cut points are singularities of the exponential map. There are typically two reasons for a cut point [47]: a conjugate point (a point where the exponential map is degenerate), and a Maxwell point (a point where two distinct geodesics meet at the same time). Finding cut points is a hard mathematical problem, and its solution relies on explicit formulas for the geodesics and analysis of symmetries of the exponential map. Thus, to solve the boundary value problems (15) and (16) of finding a length minimizer between two given configurations, one needs to restrict the preimage of the exponential map to the domain corresponding to only optimal geodesics. Further, the shooting method can be applied.

## 6. Modeling of Association Field

The problem of contour completion (integration) by human visual system was investigated by psychophysicists. Gestalt laws have been proposed for several phenomena of visual perception. Among them, the law of good continuation plays the central role for perceptual completion. The principle of good continuation is found in the experiments of Field, Hayes and Hess [51]. Those experiments have resulted in the notion of association field, which describes the set of possible subjective contours starting from a given initial configuration. The role of the scale in contour integration process was stressed in [52].

**Figure 3:**
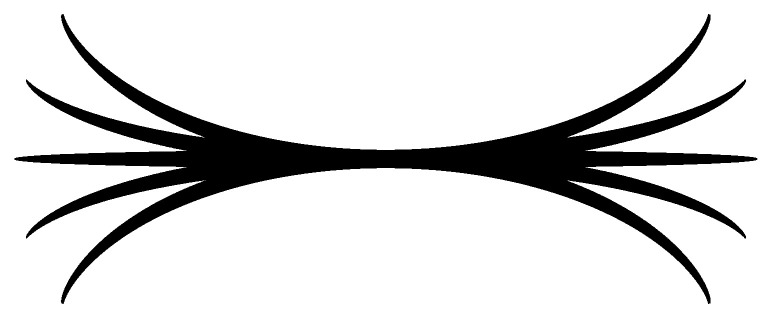
Modeling of association field by sub-Riemannian geodesics in SIM(2). The spatial projections of the geodesics are depicted for the following initial covectors: $h_{20}=h_{40}=0;\left(h_{10},h_{30}\right)\in \left\{\left(\pm 1,0\right),\left(\pm 0.93,\pm 0.35\right),\left(\pm 0.99,\pm 0.11\right)\right\}$.

Inspired by Figure 16 in [51], we provide a simulation of association field by sub-Riemannian geodesics in SIM(2) (see Figure 3). A remarkable property of this model is that the further spatial propagation of the present geodesics does not appear with growing time, which corresponds to Hypothesis 1. This gives a natural bound for the spacial distance between given boundary configurations, which corresponds to the Field model.

**Figure 4:**
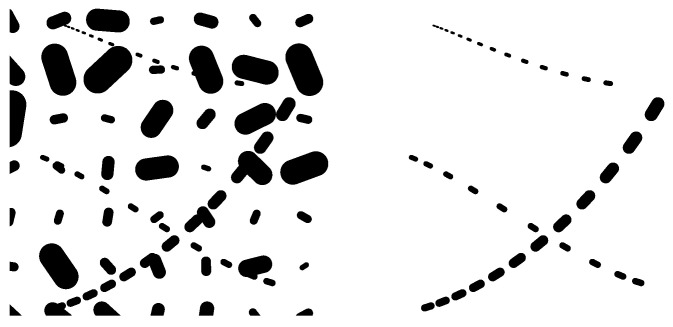
Perceptual grouping of the elements in SIM(2) having small SR distance between them. They are plotted over the background consisting of the elements located from each other on a bigger distance.

In Figure 4, we provide a simulation showing that the sub-Riemannian distance in SIM(2) can be used as a criterion for perceptual grouping of the patterns with different positions, orientations, and sizes. In this experiment, we show the points in SIM(2) that are equidistantly (with the distance d=0.1) distributed along the given three segments of geodesics. They are plotted over the background consisting of points in SIM(2) on a regular spatial (x,y) grid and with randomly chosen orientation $\theta \in S^{1}$ and the scale σ∈[0,1.8]. The grid is constructed in a way to guarantee that the distance between the background elements is greater than 0.18. One can see that the equidistantly distributed points are grouped in contrast to the points in the background.

We performed the above simulations in Wolfram Mathematica by numerical integration of the normal Hamiltonian system (33). In the experiments, we relied on the local properties of the exponential map. A further detailed study of the feasibility of SIM(2) model for contour completion on real images requires software to solve a boundary value problem, as discussed in Section 5. This will be a topic of our future research.

## 7. Conclusions

In this paper, we considered the sub-Riemannian problem in the Lie group SIM(2) of orientation-preserving similarity transformations of the plane. This problem arises when modeling the mechanism of completing contours by the visual cortex. We considered an extended Petitot–Citti–Sarti model, where the thickness of the contours is taken into account. Based on the principle of minimum energy in the contour completion process, we proposed a sub-Riemannian metric encoding the energy. We stated the contour completion problem as the problem of finding a length-minimizer with given boundary conditions. We reformulated the problem as a time-optimal problem. We proved a solution’s existence and applied a necessary optimality condition: PMP. The Hamiltonian PMP system for the geodesics was derived. We found explicit parameterization of abnormal trajectories and provided a qualitative analysis of the normal Hamiltonian system. Finally, we presented simulations on constructing the association field by sub-Riemannian geodesics in SIM(2).

## Figures and Tables

**Figure A1:**
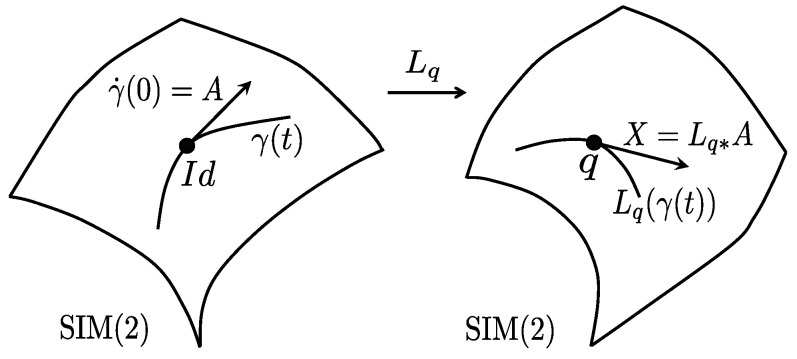
Construction of left-invariant vector fields. Here, γ is a smooth curve passing through the unit element Id, $L_{q}$ is the left translation on element q∈SIM(2), and *A* is the tangent vector of γ.

## Data Availability

Dataset available on request from the authors.

## Nomenclature

R	Set of real numbers.
Z	Set of integer numbers.
$S^{1}$	One dimensional sphere (a circle): $S^{1}=R/2\pi Z$.
SO(2)	Lie group of rotations of the plane $R^{2}$. This group is parameterized by angle $\theta \in S^{1}$.
SE(2)	Lie group of proper motions of the plane $R^{2}$. This group is topologically equivalent to the manifold $R^{2}\times S^{1}$ and parameterized by a vector of parallel translation $\left(x,y\right)\in R^{2}$ and an angle $\theta \in S^{1}$.
SIM(2)	Lie group of orientation-preserving similarity transformations of the plane $R^{2}$. Its matrix representation is given by (1).
*M*	Short notation for the Lie group SIM(2).
$\tilde{\omega }$	Differential one form in SE(2) in the classic Petitot–Citti–Sarti model.
Ker	Kernel of a differential form. It is a linear space spanned by the vectors vanishing the form.
$\tilde{\Delta }$	Distribution in TSE(2) in the classic Petitot–Citti–Sarti model.
$\tilde{g}$	Metric in SE(2) in the classic Petitot–Citti–Sarti model.
*q*	Element of SIM(2).
(x,y)	The parameters of SIM(2): $\left(x,y\right)\in R^{2}$ is a vector of parallel translation. In our model, (x,y) are coordinates in image plane.
θ	The parameter of SIM(2): $\theta \in S^{1}$ is an angle of rotation. In our model, θ is the orientation angle between the abscissa and the tangent vector to the contour.
σ	The parameter of SIM(2): σ∈R is a scaling parameter. In our model, $e^{\sigma }$ is the thickness of the contour.
Id	Unit element of the group SIM(2). It is given unit matrix and corresponds to the parameters values x=y=θ=σ=0.
*G*	The Gabor function, see (3).
*I*	Image on the retinal plane.
*O*	Lifted image in the extended space SIM(2), see (5).
$L_{q}$	Left translation on element q∈SIM(2), see (A3).
$X_{i}$	Left-invariant vector field on SIM(2), see (9)–(12).
ω	Differential one form in SIM(2), see (7).
Δ	Distribution in TSIM(2), see (8)
γ	Horizontal curve in SIM(2), see (13).
l(γ)	Sub-Riemannian length of a horizontal curve γ, see (14).
*T*	Terminal time in the optimal control problem.
*u*	The control vector.
$u_{i}$	*i*-th component of the control vector *u*.
*U*	Set of admissible controls, see (28).
[·,·]	Commutator (also known as Lie bracket). Commutator of two matrices $A_{i}$ and $A_{j}$ is defined by $\left[A_{i},A_{j}\right]=A_{i}A_{j}-A_{j}A_{i}$. Commutator of two vector fields $X_{i},X_{j}\in TM$ at a point q∈M is defined by ${\left[X_{i},X_{j}\right]}_{q}={\left.\frac{\partial }{\partial s\partial t}\right|}_{t=s=0}\;e^{-tX_{i}}\circ e^{sX_{j}}\circ e^{tX_{i}}\left(q\right)$, where $e^{tX}$ denotes a flow generated by the vector field *X*, see [47].
sim(2)	Lie algebra of the Lie group SIM(2). This is a vector space $sim(2)=T_{\mathrm{Id}}SIM(2)$ spanned by the basis vectors $A_{1},\ldots ,A_{4}$, see (A4) for their matrix representation.
Lie	Lie algebra generated by the given vector fields and all their commutators, see (18).
*p*	Adjoint covector in the Darboux coordinates.
$p_{i}$	*i*-th component of the covector *p*.
*h*	Adjoint covector in the left-invariant coordinates.
$h_{i}$	Left-invariant Hamiltonian corresponding to the basis vector field $X_{i}$, see (19). $h_{i}$ is the *i*-th component of the covector *h*.
$h_{i0}$	Initial (for t=0) value of $h_{i}$.
$h_{i1}$	Terminal (for t=T) value of $h_{i}$.
$H_{u}$	Pontryagin function, see (19).
$Y_{i}$	Right-invariant vector field.
$g_{i}$	Right-invariant Hamiltonian, see (34).
*P*	Poisson bivector.
{·,·}	Poison bracket.
〈·,·〉	Scalar product of a covector and a vector, $\langle \sum p_{i}d\;x_{i},\sum v_{i}\frac{\partial }{\partial x_{i}}\rangle =\sum p_{i}v_{i}$.

## Abbreviations

The following abbreviations are used in this manuscript:

LGN	lateral geniculate nucleus
RF	receptive fields
V1	primary visual cortex
PMP	Pontryagin maximum principle
SR	sub-Riemannian

## Appendix A. Construction of the Left-Invariant Distribution

The Lie group of orientation-preserving similarity transformations on the plane is

(A1) $$SIM(2)=\left\{q=\left.\left(\begin{array}{ccc} e^{\sigma }\cos\theta & -e^{\sigma }\sin\theta & x\\ e^{\sigma }\sin\theta & e^{\sigma }\cos\theta & y\\ 0 & 0 & 1 \end{array}\right)\;\right|\;\left(x,y\right)\in R^{2},\theta \in S^{1},\sigma \in R\right\}.$$

The basis vector fields associated with the coordinates (x,y,θ,σ) are given by

(A2) $$\begin{array}{c} \frac{\partial }{\partial x}=\begin{array}{c} \left(\begin{array}{ccc} 0 & 0 & 1\\ 0 & 0 & 0\\ 0 & 0 & 0 \end{array}\right) \end{array},\;\frac{\partial }{\partial \theta }=\begin{array}{c} \left(\begin{array}{ccc} -e^{\sigma }\sin\theta & -e^{\sigma }\cos\theta & 0\\ e^{\sigma }\cos\theta & -e^{\sigma }\sin\theta & 0\\ 0 & 0 & 0 \end{array}\right) \end{array}\;\;,\\ \frac{\partial }{\partial y}=\begin{array}{c} \left(\begin{array}{ccc} 0 & 0 & 0\\ 0 & 0 & 1\\ 0 & 0 & 0 \end{array}\right) \end{array},\;\frac{\partial }{\partial \sigma }=\begin{array}{c} \left(\begin{array}{ccc} e^{\sigma }\cos\theta & -e^{\sigma }\sin\theta & 0\\ e^{\sigma }\sin\theta & e^{\sigma }\cos\theta & 0\\ 0 & 0 & 0 \end{array}\right) \end{array}\;\;\;. \end{array}$$

Denote by Id the unit element, which is given by identity matrix and corresponds to the origin, and denote by $L_{q}$ the left translation on element q∈SIM(2), which is given by matrix multiplication

(A3) $$L_{q}q^{\prime }=qq^{\prime }.$$

The canonical basis for the Lie algebra $sim(2)=T_{\mathrm{Id}}SIM(2)$ is given by

(A4) $$A_{1}=\begin{array}{c} \left(\begin{array}{ccc} 0 & 0 & 1\\ 0 & 0 & 0\\ 0 & 0 & 0 \end{array}\right) \end{array},\;A_{2}=\begin{array}{c} \left(\begin{array}{ccc} 0 & 0 & 0\\ 0 & 0 & 1\\ 0 & 0 & 0 \end{array}\right) \end{array},\;A_{3}=\begin{array}{c} \left(\begin{array}{ccc} 0 & -1 & 0\\ 1 & 0 & 0\\ 0 & 0 & 0 \end{array}\right) \end{array},\;A_{4}=\begin{array}{c} \left(\begin{array}{ccc} 1 & 0 & 0\\ 0 & 1 & 0\\ 0 & 0 & 0 \end{array}\right) \end{array}.$$

Every left-invariant vector field is obtained by push-forward of a Lie algebra element under the left translation. The basis left-invariant vector fields are defined as $X_{i}\left(q\right)=L_{q*}A_{i}$ (see Figure A1). Explicitly, this construction is the following: Let γ(t):R→SIM(2) be a smooth curve such that γ(0)=Id, ${\left.\frac{d\;}{d\;t}\right|}_{t=0}\gamma \left(t\right)=A_{i}$. Then, $X_{i}$ is given by $X_{i}\left(q\right)={\left.\frac{d\;}{d\;t}\right|}_{t=0}L_{q}\left(\gamma \left(t\right)\right)$, or $X_{i}\left(q\right)=qA_{i}$ in matrix representation

(A5) $$\begin{array}{c} X_{1}\left(q\right)=\begin{array}{c} \left(\begin{array}{ccc} 0 & 0 & e^{\sigma }\cos\theta \\ 0 & 0 & e^{\sigma }\sin\theta \\ 0 & 0 & 0 \end{array}\right) \end{array},\;X_{3}\left(q\right)=\begin{array}{c} \left(\begin{array}{ccc} -e^{\sigma }\sin\theta & -e^{\sigma }\cos\theta & 0\\ e^{\sigma }\cos\theta & -e^{\sigma }\sin\theta & 0\\ 0 & 0 & 0 \end{array}\right) \end{array}\;\;,\\ X_{2}\left(q\right)=\begin{array}{c} \left(\begin{array}{ccc} 0 & 0 & -e^{\sigma }\sin\theta \\ 0 & 0 & e^{\sigma }\cos\theta \\ 0 & 0 & 0 \end{array}\right) \end{array},\;X_{4}\left(q\right)=\begin{array}{c} \left(\begin{array}{ccc} e^{\sigma }\cos\theta & -e^{\sigma }\sin\theta & 0\\ e^{\sigma }\sin\theta & e^{\sigma }\cos\theta & 0\\ 0 & 0 & 0 \end{array}\right) \end{array}\;\;\;. \end{array}$$

Thus, we have

(A6) $$\begin{array}{c} X_{1}\left(q\right)=e^{\sigma }\left(\cos\theta \frac{\partial }{\partial x}+\sin\theta \frac{\partial }{\partial y}\right),\;X_{3}\left(q\right)=\frac{\partial }{\partial \theta },\\ X_{2}\left(q\right)=e^{\sigma }\left(-\sin\theta \frac{\partial }{\partial x}+\cos\theta \frac{\partial }{\partial x}\right),\;X_{4}\left(q\right)=\frac{\partial }{\partial \sigma }. \end{array}$$
